# Marfan Syndrome Variability: Investigation of the Roles of Sarcolipin and Calcium as Potential Transregulator of *FBN1* Expression

**DOI:** 10.3390/genes9090421

**Published:** 2018-08-21

**Authors:** Louise Benarroch, Mélodie Aubart, Marie-Sylvie Gross, Marie-Paule Jacob, Pauline Arnaud, Nadine Hanna, Guillaume Jondeau, Catherine Boileau

**Affiliations:** 1 Laboratory for Vascular Translational Science, INSERM U1148, Centre Hospitalo-Universitaire Xavier Bichat, 46 rue Henri Huchard, 75018 Paris, France; louise.benarroch@inserm.fr (L.B.); melodie.aubart@inserm.fr (M.A.); marie-sylvie.gross@inserm.fr (M.-S.G.); marie-paule.jacob-lenet@inserm.fr (M.-P.J.); pauline.arnaud@inserm.fr (P.A.); guillaume.jondeau@aphp.fr (G.J.); 2 Service de Neuropédiatrie, Hôpital Necker-Enfants-Malades, 149 rue de Sèvres, 75015 Paris, France; 3 Département de Génétique, Centre Hospitalo-Universitaire Xavier Bichat, 46 rue Henri 17 Huchard, 75018 Paris, France; nadine.hanna@aphp.fr; 4 Centre de Référence pour le Syndrome de Marfan et Syndromes Apparentés, Service de Cardiologie, Centre Hospitalo-Universitaire Xavier Bichat, 46 rue Henri Huchard, 75018 Paris, France

**Keywords:** Marfan syndrome, variability, fibrillin-1, calcium, sarcolipin, genetic modifiers

## Abstract

Marfan syndrome (MFS) is an autosomal dominant connective tissue disorder that displays a great clinical variability. Previous work in our laboratory showed that fibrillin-1 (*FBN1*) messenger RNA (mRNA) expression is a surrogate endpoint for MFS severity. Therefore, an expression quantitative trait loci (eQTL) analysis was performed to identify trans-acting regulators of *FBN1* expression, and a significant signal reached genome-wide significant threshold on chromosome 11. This signal delineated a region comprising one expressed gene, *SLN* (encoding sarcolipin), and a single pseudogene, *SNX7-ps1* (CTD-2651C21.3). We first investigated the region and then looked for association between the genes in the region and *FBN1* expression. For the first time, we showed that the *SLN* gene is weakly expressed in skin fibroblasts. There is no direct correlation between *SLN* and *FBN1* gene expression. We showed that calcium influx modulates *FBN1* gene expression. Finally, *SLN* gene expression is highly correlated to that of the neighboring *SNX7-ps1*. We were able to confirm the impact of calcium influx on *FBN1* gene expression but we could not conclude regarding the role of sarcolipin and/or the eQTL locus in this regulation.

## 1. Introduction

Marfan syndrome (MFS; OMIM #154700) is an autosomal dominant connective tissue disorder with an estimated prevalence of 1/5000 individuals. It is a multisystemic disease that affects the ocular, skeletal, and cardiovascular system as well as lung, skin, and dura. Thoracic aortic aneurysm (TAA) is the main cardiovascular feature that can lead to dissection or rupture of the aortic wall, the major life-threatening event in MFS. In most cases, MFS is due to mutation in the *FBN1* gene encoding fibrillin-1, an extracellular matrix protein. To date, over 3000 mutations have been reported in the *FBN1* database [1,2].

The syndrome displays great clinical variability, regarding age of onset and number and severity of the symptoms that cannot be explained by the few genotype-phenotype correlations identified between *FBN1* mutations and MFS. Furthermore, this variability is observed not only between families, but also among relatives. Genotype-phenotype correlation [3,4,5] could explain interfamilial variability but not the intrafamilial variability, which suggests the existence of genetic factors underlying phenotypic variability.

Recently, our team investigated the hypothesis that phenotype severity could be related to the variable expression level of *FBN1* synthesized from the wildtype (WT) allele [6]. *FBN1* gene expression levels were assessed in culture of skin fibroblasts from 80 controls and from 80 MFS patients with premature termination codon (PTC) mutations. Results in controls showed a 3.9-fold variation in *FBN1* messenger RNA (mRNA) synthesis level between controls. A similar 4.4-fold variation was found in the MFS population, but the mean level of *FBN1* expression level was half of the control population. Differential allelic expression analysis in MFS fibroblasts showed that no residual expression of the mutated allele was detected. Moreover, a low level of residual WT *FBN1* mRNA accounted for a high risk of ectopia lentis and pectus abnormality and tended to increase the severity of aortic dilatation. In the control population, independently of the expression level of *FBN1*, we observed a steady-state equilibrium between the two allelic expressions, suggesting that the *FBN1* expression level mainly depends on trans-acting regulators [6].

An expression quantitative trait loci (eQTL) analysis was performed to identify trans-acting regulators of *FBN1* expression, in PTC-mutation carriers. A significant signal that reached genome-wide significant threshold was identified on chromosome 11 (*p* = 6 × 10^−8^) at 11q22.3 (gMod-M3) [5]. This trans-eQTL was located in a linkage disequilibrium (LD) block and delineating a 40 kb region containing only one expressed gene, *SLN* (encoding sarcolipin), and a single pseudogene, *SNX7-ps1* (CTD-2651C21.3) [5]. Since the lead single nucleotide polymorphism (SNP) had never been highlighted in any reported genome-wide study, we first investigated the region and then looked for association between the genes in the region and *FBN1* expression.

## 2. Materials and Methods

### 2.1. Patient and Control Samples

Marfan syndrome patients were recruited in the ‘Centre National Maladies Rares—Syndrome de Marfan et apparentés’, the French National Reference Centre located at Bichat Hospital (Paris, France). Clinical diagnosis and systemic score were established according to the revised Ghent nosology as already reported in Aubart et al [5]. For the purpose of this study, skin fibroblasts from 40 MFS patients carrying a PTC-*FBN1* mutation were selected according to their genotype at rs11212346:20 with [CC] genotype, 19 with [CT] genotype, and 1 with [TT] genotype. All patients with a [CT] genotype and [TT] genotype were selected and matched with 20 [CC] genotype patients. To develop and validate the various tests, three control skin fibroblasts were used.

All patients originated from the French National Reference Centre for Marfan Syndrome and related disorders and gave their written informed consent for participation in this clinical and genetic study in agreement with the requirements of French regulations (Accepted by “Comite’ de Protection des Personnes CPP Ile de France XI”, 78105 St Germain en Laye; with the registration number #11008).

### 2.2. Cell Culture and RNA Purification

Patient skin fibroblasts were cultivated in DMEM (Thermo Scientific, Villebon sur Yvette, France) supplemented with 4.5 g/L glucose, 15% Fetal Bovine Serum (FBS, PAA Laboratory, Villacoublay, France), and antibiotics (50 U/mL of penicillin, streptomycin, and amphotericin B) (PAA Laboratory, Villacoublay, France) as previously described [6].

From fibroblast culture, total RNAs were extracted with the miRNeasy kit^®^ (Qiagen S.A., Courtaboeuf, France) according to the manufacturer’s instructions. After purification, RNAs were eluted in 20 µL of RNase-free water and RNA concentrations were estimated by measuring absorbance at 260 nm, using Nanodrop 2000/2000 c system (Thermo Scientific, Villebon sur Yvette, France).

### 2.3. Digital Droplet PCR (ddPCR)

To assess expression level of low-expressed genes, a digital droplet PCR (ddPCR) was performed. Reverse transcription was performed using miScript II RT kit (Qiagen S.A., Courtaboeuf, France), according to the manufacturer’s recommendations. Droplet digital PCR was performed at a concentration of 50 ng/µL per well. For each assay, the gene of interest (*SLN* or *SNX7-ps1*) and control gene (*SDHA*) were run in multiplex using 6-carboxyfluoresceine (FAM) and hexachloro-fluorescein (HEX) labeling, respectively. Reaction mixtures were prepared using ddPCR™ Supermix for Probes (BioRad, Marnes-la-Coquette, France), DNA, and PrimePCR Probe Assay (BioRad, Marnes-la-Coquette, France) according to the manufacturer’s recommendations. Droplets were generated from the mixtures using Droplet Generation Oil for Probes (BioRad, Marnes-la-Coquette, France) on the QX100 Droplet Generator (BioRad, Marnes-la-Coquette, France) according to the manufacturer’s instructions. PCR was carried out on a thermal cycler for 40 cycles using a 55 °C annealing temperature and fluorescent level of each droplet was read using the QX100 Droplet Reader (BioRad, Marnes-la-Coquette, France). Data were analyzed and the concentration of mRNA levels for both genes and the ratio of gene of interest/reference gene were calculated using QuantaSoft software (BioRad, Marnes-la-Coquette, France). Each DNA sample was run in two technical replicates and three biological replicates.

### 2.4. Real-Time Quantitative PCR (RT-qPCR)

Reverse transcription was performed using miScript II RT kit, according to the manufacturer’s recommendations. The final concentration of complementary DNA (cDNA) was 100 ng/µL for each sample. RT-qPCR was performed using ABI PRISM 7300 (Applied Biosystems, Thermo Scientific, Waltham, MA, USA), according to the manufacturer’s instructions. Absolute Blue qPCR SYBR Green supermix (Thermo Scientific, Villebon sur Yvette, France) and specific primers (Table S1) were used to quantify *FBN1*, *SNX7*, *FOS*, *SDHA*, and *GAPDH* gene expression. Results were then normalized with the expression of the two housekeeping genes (*SDHA* and *GAPDH*) by calculating the geometric means [7].

### 2.5. Cell Culture and Pharmacological Treatment with Calcium Ionophore, A23187

For the pharmacological treatment, 7 × 10^5^ skin fibroblasts were seeded in each well of 4-well plates (21.8 cm^2^/well). Cells were incubated with A23187, a calcium ionophore (Sigma-Aldrich, Darmstadt, Germany). Each cell preparation was treated with two doses of A23187, 1 µM and 10 µM, in duplicates and at three different times of incubation (4, 24, and 48 h). Skin fibroblasts were deprived of FBS and antibiotics 24 h before treatment. A23187 was solubilized in DMSO and diluted in FBS-free DMEM before its addition to cells. In control wells, a similar volume of DMSO, diluted in FBS-free DMEM, was added. Expression levels of *FOS* gene were quantified as a positive control for the entry of calcium in the cells [8].

### 2.6. Mimic and micro RNA (miRNA) Transfection

TargetScan [9] was used to predict biological targets of micro RNAs (miRNAs) by searching for the presence of conserved sequenced corresponding to miRNA binding site. P_CT_ (probability of conserved targeting) estimates the probability of the site being preferentially conserved because it is targeted by the cognate miRNA [10].

For the transfection assay, 6 × 10^5^ skin fibroblasts were seeded in each well of 4-well plates (21.8 cm^2^/well). Transfection was performed using Hiperfect Transfection Reagent (Qiagen S.A., Courtaboeuf, France), 150 ng miScript miRNA Mimic (Syn-hsa-miR-9-5) (Qiagen S.A., Courtaboeuf, France) or 1500 ng miScript miRNA Inhibitor (Anti-hsa-miR-9-5) (Qiagen S.A., Courtaboeuf, France), according to the manufacturer’s recommendation. For controls, transfection control (Hiperfect Transfection Reagent only) and a negative control (1500 ng miScript Inhibitor Negative Control; Qiagen S.A., Courtaboeuf, France) had been tested. Each condition had been tested in three biological replicates.

Total RNA were extracted 72 h after transfection with the miRNeasy kit^®^ according to the manufacturer’s instructions. After purification, RNAs were eluted in 15 µL of RNase-free water and the concentrations of RNA were estimated by measuring absorbance at 260 nm, using Nanodrop 2000/2000 c system. Reverse transcription was performed as above.

### 2.7. Statistical Analysis

Statistical analysis was performed using GraphPad Prism version 7.0 software. The statistical difference between two groups was tested using nonparametric Mann–Whitney *U* test, and between three groups using One Way ANOVA or Kruskall–Wallis test. The coefficient of correlation (*r*^2^) was calculated by linear regression and the statistical significance by Spearman correlation.

## 3. Results

### 3.1. Investigation of the 11q22.3 Region

**Figure 1:**
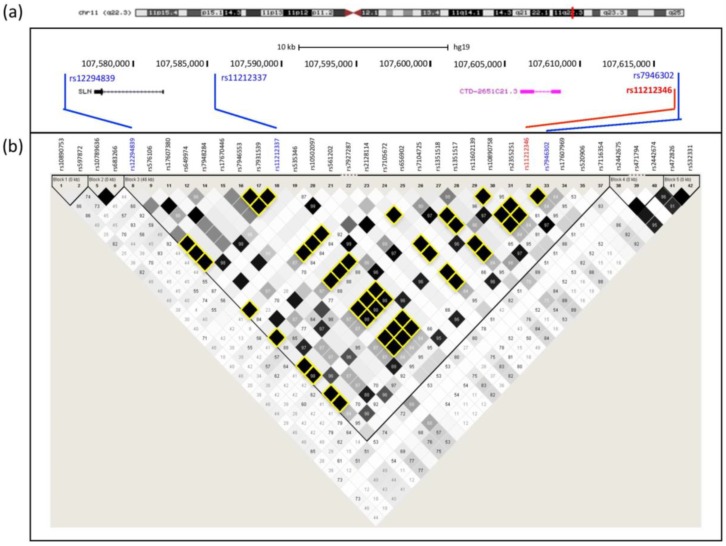
Linkage disequilibrium (LD) block for (fibrillin-1) *FBN1* expression transregulator region at 11q22.3: (**a**) Cytogenetic localization and sequence coordinates of lead single nucleotide polymorphisms (SNPs) in red; (**b**) Regional LD map. Data were extracted from HapMap using Haploview. Linkage disequilibrium was measured as *r^2^* values which range from 0 (no correlation) to 1 (complete correlation). SNPs in complete LD (*r^2^* = 1) with SNPs identified by eQTL analysis are highlighted in yellow.

The lead SNP at 11q22.3, rs11212346 (chr11:107617257C > T; *p*-value = 6 × 10^−8^), as well as three other SNPs [rs12294839 (chr11:107576185T > G), rs1121337 (chr11:107586240G > A), and rs7946302 (chr11:107617516C > T)] are in complete LD (Figure 1a). Six others are also in the HapMap LD block (Table 1), which delineates a 40 kb region. This region contains only one expressed gene, *SLN*, and a single pseudogene, *SNX7-ps1* (CTD-2651C21.3) (Figure 1b).

The *SLN* gene encodes sarcolipin, a 31-amino-acid protein, highly expressed in skeletal muscle and at a lower extent in the atria [11]. Sarcolipin inhibits the activity of cardiac sarco(endo)plasmic reticulum Ca^2+^-ATPase 1 (SERCA1) pumps in cardiac and skeletal muscle by decreasing their apparent affinity for Ca^2+^ [12]. The presence of a potential functional variant in *SLN* responsible for the variability of expression was investigated. Systematic sequencing of the single exon in this gene was performed in the 40 genotyped MFS patients. Not a single variant, heterozygous or homozygous, was found. Since the sequence of the pseudogene *SNX7-ps1* shares great homology (92%) with that of its parent gene, *SNX7*, it could not be sequenced.

**Figure 2:**
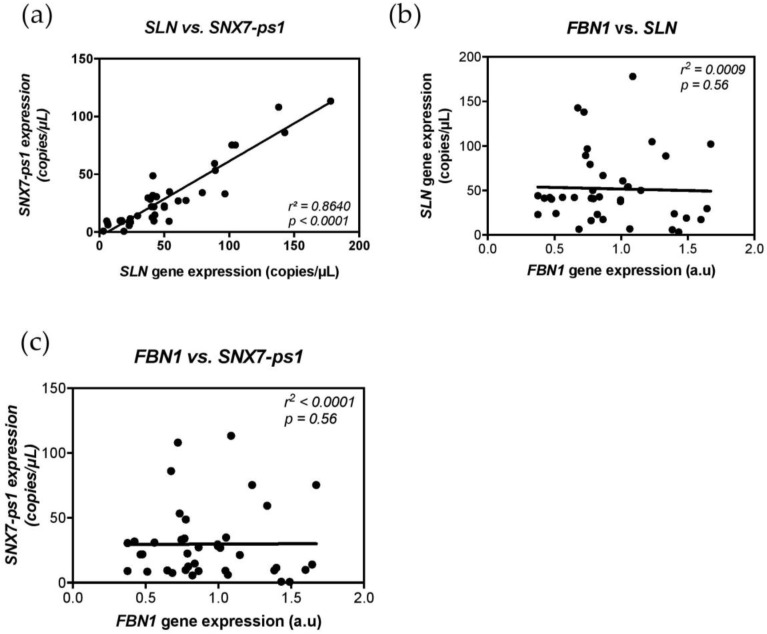
Correlation of expression levels between *FBN1 SLN* and *SNX7-ps1* gene. (**a**) Gene expression levels were assessed using digital droplet PCR (ddPCR). Positive correlation of expression was observed between the nearest gene of the lead SNP, *SLN* and *SNX7-ps1* (*r*^2^ = 0.86, *p*-value < 0.0001). (**b**) Correlation plot between *FBN1* and *SLN* expression. No correlation was found (*r*^2^ = 0.0009, *p*-value = 0.56). (**c**) Correlation plot between *FBN1* and *SNX7-ps1* expression. No correlation was found (*r*^2^ < 0.0001, *p*-value = 0.56).

One of the SNPs in the LD block, rs7104725, is located in the pseudogene coding sequence and could be involved in a gene expression regulatory mechanism. Therefore, we assessed gene expression of *SLN* and *SNX7-ps1*, for which no information was available for our cell models (skin and adventitial fibroblasts). Furthermore, it was unknown whether the pseudogene was expressed. Droplet digital PCR was used to investigate *SLN* and *SNX7-ps1* expression levels on skin fibroblasts from 40 MFS patients. The results showed that indeed it was expressed, that expression levels for both genes were very low, and that they were correlated (Figure 2a).

Gene expression levels for *SLN* and *SNX7-ps1* based on rs11212346 genotype groups were investigated. No differences were observed and we excluded a local regulation on *SLN* or *SNX7-ps1* expression by rs11212346 or other SNPs in complete LD (Figure S1). To investigate the potential role of *SLN* on *FBN1* gene regulation, a correlation between *SLN* and *FBN1* gene expression was assessed. No correlation of expression was found, excluding *SLN* as a direct modifier of *FBN1* expression (Figure 2b).

We hypothesized that *SNX7-ps1* could be an antisense regulator of *FBN1* gene expression but no correlation was found between *SNX7-ps1* and *FBN1* gene expressions, excluding *FBN1* as a direct target of the pseudogene (Figure 2c). We also investigated a potential role of the parent gene *SNX7* as an intermediate regulator between *SNX7-ps1* and *FBN1* gene regulation. Despite a visibly significant increase of *SNX7* expression found for the single homozygous [TT] subject, no correlation was found between the expression of *FBN1*, *SNX7-ps1*, and *SNX7* (Figure S2).

A study on a possible role of *SNX7-ps1* as a miRNA decoy for *FBN1* and *SNX7* miRNA binding was investigated. Indeed, in silico analysis of *SNX7* gene sequence using Targetscan [9] showed a potential binding site for miR9 (hsa-mir-9) on the 3′-UTR region of *SNX7* (score = 0.61). In silico analysis also predicted *FBN1* as a potential target of miR9, with a high binding affinity (score = 0.97). In 13 MFS patient skin fibroblasts, we transfected small interfering RNA (siRNA) against miR9 (inhibitor) as well as a synthetic miRNA (Mimic) of miR9 for 72 h. No significant difference for *FBN1* gene expression was observed (*p*-value = 0.07). However, we noted a trend of a decreased *FBN1* expression in the presence of the synthetic miR9 and an increased *FBN1* expression with an excess of miR9 (Figure S3). However, data were not conclusive regarding *SNX7* gene expression or *SNX7-ps1* expression (Figure S3). Hence, miR9 may have an impact on *FBN1* gene expression independently of *SNX7-ps1* and *SNX7* gene. Taken together, all of these results excluded *SNX7-ps1* as well as *SNX7* as modifiers of *FBN1* gene expression.

### 3.2. Sarcolipin and Calcium Impact on *FBN1* Gene Expression

**Figure 3:**
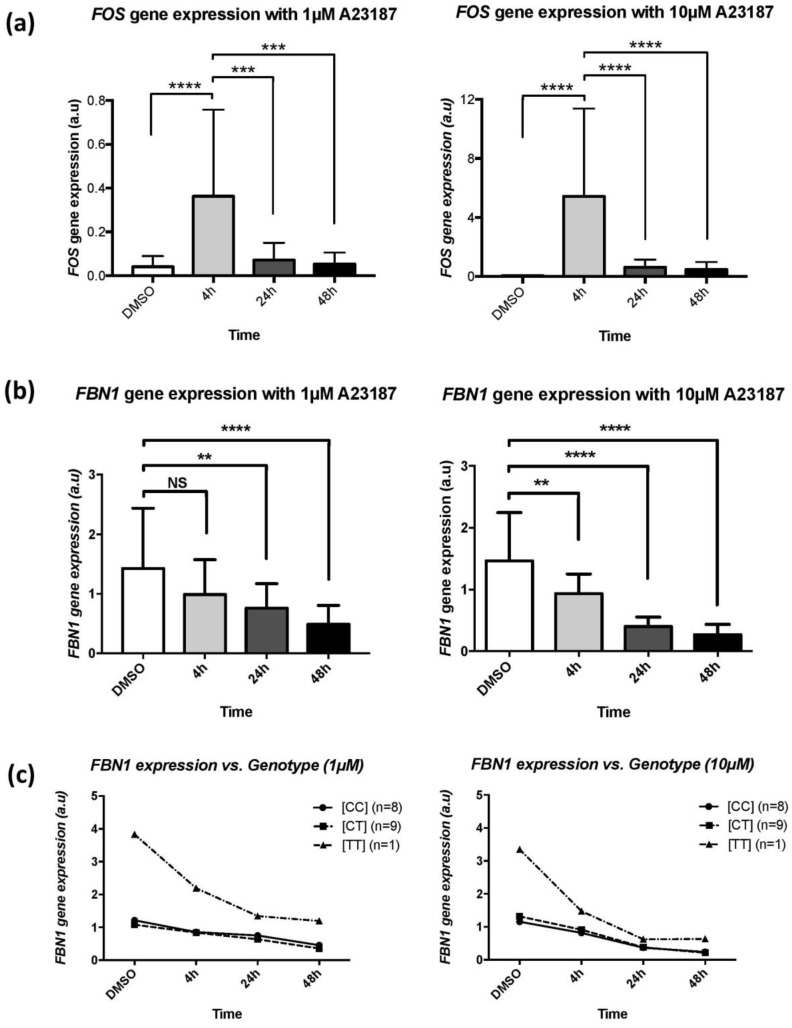
Effect of A23187 on gene expression. Patients’ skin fibroblasts were incubated with DMSO (untreated), 1 µM or 10 µM of A23187 for 4 h, 24 h, and 48 h. (**a**) *FOS* gene expression. (**b**) *FBN1* gene expression after treatment with A23187 (Control vs. 4 h: *p*-value = 0.0082; Control vs. 24 h: *p*-value < 0.0001; Control vs. 48 h: *p*-value < 0.0001). (**c**) *FBN1* gene expression according to rs11212346 genotype (Control vs. 4 h: *p*-value = 0.0906; Control vs. 24 h: *p*-value = 0.0075; Control vs. 48 h: *p*-value < 0.0001). Genotype: [CC] (n= number of subject); [CT] (n); [TT] (n). [NS] *p*-value > 0.05; ** *p*-value < 0.01; *** *p*-value < 0.001; **** *p*-value < 0.0001.

Sarcolipin is known to be a regulator of Ca^2+^ homeostasis in the cells. Hence, we hypothesized that Ca^2+^ could be an intermediate regulator between *SLN* and *FBN1* gene expression. We tested the effect of calcium on *FBN1* gene expression in skin fibroblasts of 18 MFS patients ([CC], *n* = 8; [CT], *n* = 9; [TT], *n* = 1) and then looked for a different regulation based on genotype of rs11212346. We treated cells with two doses of A23187, 10 µM and 1 µM at different time points (4, 24, and 48 h). After treatment of 4 h with 1 or 10 µM of A23187, we observed a significant increase of *FOS* expression for both doses compared to untreated cells (*p*-value < 0.0001) followed by a return to normal after 24 and 48 h, which attested to calcium entry in the cell (Figure 3a). From 4 to 48 h after treatment with 10 µM of A23187, we observed a significant decrease of *FBN1* gene expression compared to control (*p*-value < 0.01) (Figure 3b). A similar variation of expression was observed after a treatment with 1 µM of A23187. At 24 and 48 h of treatment, *FBN1* expression was significantly decreased (*p*-value < 0.01) (Figure 3b). Similar results were obtained in control skin fibroblasts (Figure S4). We concluded that free intracellular concentration of calcium regulates *FBN1* gene expression in a dose-dependent manner in skin fibroblasts of *FBN1* PTC-mutation carriers.

To test sarcolipin as an effector in this regulation, we compared *FBN1* expression for the different genotypes. Despite decreased *FBN1* expression, there was no difference between [CC] and [CT] subjects (Figure 3c). The homozygous [TT] subject displayed a different response that needs to be confirmed in other [TT] subject. Taken together, these results showed that calcium has an effect on *FBN1* expression that seems to be independent of *SLN* expression.

## 4. Discussion

This work was aimed at investigating the mechanism underlying the trans-eQTL signal (*p* = 6 × 10^−8^) for *FBN1* expression in fibroblast on chromosome 11. This trans-eQTL is located in an LD block, which contains only one expressed gene, *SLN*, and a single pseudogene, *SNX7-ps1*. Since the lead SNP (rs11212346) had never been highlighted in any reported genome-wide study, we first investigated the region and then looked for association between the genes in the region and *FBN1* expression. We showed that the *SLN* gene is weakly expressed in skin fibroblasts in controls and in MFS subjects. We also showed that the pseudogene is expressed and that expressions of *SLN* and *SNX7-ps1* are correlated. To our knowledge, this has never been reported before for these two genes even if the mechanism has been described [13]. Furthermore, since we have shown that skin fibroblasts are a surrogate for adventitial fibroblasts [6], it is highly probable that this observation extends to this cell type.

The presence of a potential functional variant in *SLN* responsible for the variability of *FBN1* expression was investigated but not found. One of the SNPs in the LD block, rs7104725, is located in the pseudogene coding sequence and could be involved in a gene expression regulatory mechanism.

We confirmed in skin fibroblasts that *SNX7-ps1* is an expressed pseudogene with a positive correlation between the *SLN* gene and *SNX7-ps1*, suggesting the role of a common regulatory element on their expression. Functional implication of pseudogenes in physiological processes is still poorly understood. However, they are known to possibly act as regulatory antisense transcripts or miRNA decoys and we investigated these two possible mechanisms [14].

Pseudogenes can function as antisense transcript, either by direct hybridization on its parent gene or by generating siRNA leading to the repression of the targeted gene expression [14]. The absence of homologous sequences and no correlation of expression between *SNX7-ps1* and the *FBN1* gene exclude *FBN1* as a direct target of *SNX7-ps1*. However, downstream target of a regulatory element such as transcription factor can establish eQTL association with the same locus if the effect is strong enough [15]. Therefore, a downstream target of *SNX7-ps1* could be responsible for *FBN1* gene regulation. We hypothesized that the *SNX7* gene could be an *FBN1* gene regulator. In our cohort, we found no correlation between *SNX7-ps1* and *SNX7* gene expression, suggesting that *SNX7-ps1* has no regulation function on *SNX7*. Moreover, the absence of correlation between rs11212346 genotype and *SNX7* as well as between *SNX7* and *FBN1* expression did not support a role of *SNX7* in *FBN1* gene regulation.

Another potential mechanism is the ability of processed pseudogenes to interfere with factors of mRNA stability. They can act as miRNA decoy: due to sequence homology between pseudogenes and miRNA target, pseudogenes can serve as bait and can compete with mRNA target for miRNA silencing [14,16]. In silico analysis demonstrated complementarity binding site between miR9, *SNX7*, *SNX7-ps1*, and *FBN1*, placing miR9 as a potential common regulator. Therefore, we investigated a potential role of miR9 in *FBN1* gene regulation. The absence of correlation between miR9 and *SNX7*, *SNX7-ps1* or *FBN1* expression did not support the role of miR9. However, miR9 is weakly expressed in skin fibroblasts and this could prevent us from observing any correlation. To counter this limitation, we transfected patients’ skin fibroblasts with a synthetic miR9 or an siRNA-targeting miR9. No difference of expression was observed for *SNX7* or *SNX7-ps1* in the different transfection conditions. Regarding *FBN1* gene expression, no significant difference was observed, but a trend with a decreased expression in the presence of miR9 and an increased expression in the absence of miR9 was observed. Moreover, a recent study showed that a down-regulation of miR9 has been associated with an increased expression level of *MMP13*, a matrix metalloproteinase that degrades collagen and other matrix components [17]. This study implicates miR9 in the extracellular matrix stability, known to be important for *FBN1* microfibrils’ proper assembly [18]. Therefore, miR9 may have an impact on *FBN1* gene regulation independently of the eQTL region.

Sarcolipin is a 31-amino-acid protein, highly expressed in skeletal muscle and at a lower extent in the atria [11]. Sarcolipin inhibits the activity of SERCA1 pumps in cardiac and skeletal muscle by decreasing its apparent affinity for Ca^2+^ [12]. During Ca^2+^ transport, SERCA transfers two molecules of Ca^2+^ per molecule of ATP hydrolysed and this process contributes to muscle thermogenesis [19,20]. Sarcolipin is a regulator of Ca^2+^ homeostasis, playing a critical role in cardiac contractility and cardiac pathophysiology [21]. Based on all of this information, the absence of a direct action of *SLN* on *FBN1* gene expression was not completely surprising. Indeed, there is no evidence in the literature that sarcolipin has a transcription factor activity giving it the ability to directly regulate gene expression. However, due to sarcolipin’s critical role on Ca^2+^ bioavailability, we hypothesized that sarcolipin could indirectly impact *FBN1* gene expression by altering intracellular Ca^2+^ homeostasis. To support the idea, Lannoy et al. showed that an increase of intracellular free calcium concentration, [Ca^2+^]_i_, had an effect on gene expression of several genes encoding for constitutive proteins of elastic fibers or involved in elastic fiber stability [22]. By using a Ca^2+^ ionophore, A23187, they increased [Ca^2+^]_i_ in vascular smooth muscle cells of Brown Norway rats and assessed its impact on the gene expression pattern. A decrease in elastic mRNA content was associated with the increase of [Ca^2+^]_i_ and the same pattern was observed for several elastic fiber-associated proteins, such as fibrillin-1. Indeed, the increase of [Ca^2+^]_i_ decreased *FBN1* gene expression in a significant manner. Moreover, Doyle et al [23] investigated the impact of calcium channel blockers (CCB), an antihypertensive compound, on TAA expansion. They showed that *Fbn1*^*C1039G*/−^ mice (modelling MFS) treated with CCB had accelerated TAA expansion and rupture through a protein kinase C-mediated pathway. In a retrospective study on patients with MFS treated with CCB, they showed that MFS patients displayed an increased risk of aortic dissection compared to patients treated with other antihypertensive compounds. These results support a cross-talk between calcium signalling, TGFβ, and aneurysms [23]. Our findings in human dermal fibroblasts extracted from MFS patients support the results of Lannoy et al [22]. Indeed, we observed a significant decrease of *FBN1* expression, in a time- and dose-dependant manner, confirming the role of calcium on *FBN1* gene regulation.

The modification of *FBN1* expression was also observed for all MFS patients with comparable pattern for the rs11212346 [CC] and [CT] genotype subjects. Interestingly, the homozygous [TT] patient displayed a much stronger response to calcium cellular influx. Therefore, it could be speculated that homozygote [TT] subjects display a possible inappropriate answer to calcium intracellular modifications. A targeted recruitment of patients with this specific genotype is warranted to confirm this result. Overall, we were able to confirm the impact of [Ca^2+^]_i_ on *FBN1* gene expression but we could not conclude regarding the role of sarcolipin and/or the eQTL locus in this regulation.

In conclusion, our data provide insight into the expression pattern of the *SLN* and *SNX7-ps1* genes at 11q22.3 but further work is necessary to unravel the mechanism underlying the trans-eQTL signal for *FBN1* expression in fibroblasts. Indeed, there is the possibility that the lead SNP could be in LD with functional variants or a regulatory SNP outside of the LD block. Finally, another avenue of research could be the investigation at the chromatin level of the area.

## Figures and Tables

**Table 1:** Single nucleotide polymorphisms (SNPs) in linkage disequilibrium at the locus 11q22.3.

SNP	Chr	Position (hg19, bp)	Major Allele	Minor Allele	MAF	LD (*r*^2^)
rs11212346	11	107,617,256	C	T	0.21	-
rs12294839	11	107,576,185	T	G	0.39	1
rs11212337	11	107,586,240	G	A	0.21	1
rs7946302	11	107,617,516	C	T	0.21	1
rs10890758	11	107,615,246	C	T	0.21	1
rs7104725	11	107,607,408	C	T	0.21	1
rs11602139	11	107,614,851	C	T	0.23	1
rs7931539	11	107,585,820	A	G	0.21	1
rs7946553	11	107,585,727	T	C	0.23	1
rs2128114	11	107,600,281	T	A	0.23	1

Red: lead SNP; Blue: SNPs identified by eQTL analysis; Black: SNPs in LD with the lead SNP; Chr: chromosome; bp: base pair; MAF: minor allele frequency. LD (*r^2^*): Linkage disequilibrium score with the lead SNP.

## Supplementary Materials

The following are available online at http://www.mdpi.com/2073-4425/9/9/421/s1: Table S1: Primers for gene expression quantification; Figure S1: Boxplot of *SLN* and *SNX7-ps1* expression according to rs11212346 genotypes; Figure S2: Correlation plot between *FBN1* and *SLN* and *SNX7-ps1* expression level; Figure S3: Exploration of the antisense hypothesis for *SNX7-ps1*, Figure S4; Effect of miR9 on gene expression.

## References

[1] Universal Mutation Database (UMD)-*FBN1*. http://www.umd.be/FBN1/orhttp://www.umd.be/FBN1/4DACTION/w_mutations.

[2] Collod-Béroud G., Béroud C., Adès L., Black C., Boxer M., Brock D.J., Godfrey M., Hayward C., Karttunen L., Milewicz D. (1997). Marfan Database (second edition): Software and database for the analysis of mutations in the human *FBN1* gene. Nucleic Acids Res..

[3] Faivre L., Collod-Beroud G., Loeys B.L., Child A., Binquet C., Gautier E., Callewaert B., Arbustini E., Mayer K., Arslan-Kirchner M. (2007). Effect of mutation type and location on clinical outcome in 1,013 probands with Marfan syndrome or related phenotypes and *FBN1* mutations: An international study. Am. J. Hum. Genet..

[4] Becerra-Muñoz V.M., Gómez-Doblas J.J., Porras-Martín C., Such-Martínez M., Crespo-Leiro M.G., Barriales-Villa R., de Teresa-Galván E., Jiménez-Navarro M., Cabrera-Bueno F. (2018). The importance of genotype-phenotype correlation in the clinical management of Marfan syndrome. Orphanet J. Rare Dis..

[5] Aubart M., Gazal S., Arnaud P., Benarroch L., Gross M.-S., Buratti J., Boland A., Meyer V., Zouali H., Hanna N. (2018). Association of modifiers and other genetic factors explain Marfan syndrome clinical variability. Eur. J. Hum. Genet..

[6] Aubart M., Gross M.-S., Hanna N., Zabot M.-T., Sznajder M., Detaint D., Gouya L., Jondeau G., Boileau C., Stheneur C. (2015). The clinical presentation of Marfan syndrome is modulated by expression of wild-type *FBN1* allele. Hum. Mol. Genet..

[7] Vandesompele J., De Preter K., Pattyn F., Poppe B., Van Roy N., De Paepe A., Speleman F. (2002). Accurate normalization of real-time quantitative RT-PCR data by geometric averaging of multiple internal control genes. Genome Biol..

[8] Rosenfeldt H., Lee D.J., Grinnell F. (1998). Increased *c-fos* mRNA expression by human fibroblasts contracting stressed collagen matrices. Mol. Cell. Biol..

[9] TargetScanHuman 7.1. www.targetscan.orgorhttp://www.targetscan.org/vert_71/.

[10] Agarwal V., Bell G.W., Nam J.-W., Bartel D.P. (2015). Predicting effective microRNA target sites in mammalian mRNAs. Elife.

[11] Odermatt A., Taschner P.E., Scherer S.W., Beatty B., Khanna V.K., Cornblath D.R., Chaudhry V., Yee W.C., Schrank B., Karpati G. (1997). Characterization of the gene encoding human sarcolipin (*SLN*), a proteolipid associated with SERCA1: Absence of structural mutations in five patients with Brody disease. Genomics.

[12] Odermatt A., Becker S., Khanna V.K., Kurzydlowski K., Leisner E., Pette D., MacLennan D.H. (1998). Sarcolipin regulates the activity of SERCA1, the fast-twitch skeletal muscle sarcoplasmic reticulum Ca^2+^-ATPase. J. Biol. Chem..

[13] Pink R.C., Wicks K., Caley D.P., Punch E.K., Jacobs L., Carter D.R.F. (2011). Pseudogenes: Pseudo-functional or key regulators in health and disease?. RNA.

[14] Li W., Yang W., Wang X.-J. (2013). Pseudogenes: Pseudo or real functional elements?. J. Genet. Genom..

[15] Michaelson J.J., Loguercio S., Beyer A. (2009). Detection and interpretation of expression quantitative trait loci (eQTL). Methods.

[16] Tutar Y. (2012). Pseudogenes. Comp. Funct. Genom..

[17] Zhang H., Song B., Pan Z. (2018). Downregulation of microRNA-9 increases matrix metalloproteinase-13 expression levels and facilitates osteoarthritis onset. Mol. Med. Rep..

[18] Sabatier L., Chen D., Fagotto-Kaufmann C., Hubmacher D., McKee M.D., Annis D.S., Mosher D.F., Reinhardt D.P. (2009). Fibrillin assembly requires fibronectin. Mol. Biol. Cell..

[19] Bombardier E., Smith I.C., Vigna C., Fajardo V.A., Tupling A.R. (2013). Ablation of sarcolipin decreases the energy requirements for Ca^2+^ transport by sarco(endo)plasmic reticulum Ca^2+^-ATPases in resting skeletal muscle. FEBS Lett..

[20] Sahoo S.K., Shaikh S.A., Sopariwala D.H., Bal N.C., Bruhn D.S., Kopec W., Khandelia H., Periasamy M. (2015). The N-terminus of sarcolipin plays an important role in uncoupling sarco-endoplasmic reticulum Ca^2+^-ATPase (SERCA) ATP hydrolysis from Ca^2+^ transport. J. Biol. Chem..

[21] Babu G.J., Bhupathy P., Timofeyev V., Petrashevskaya N.N., Reiser P.J., Chiamvimonvat N., Periasamy M. (2007). Ablation of sarcolipin enhances sarcoplasmic reticulum calcium transport and atrial contractility. Proc. Natl. Acad. Sci. USA.

[22] Lannoy M., Slove S., Louedec L., Choqueux C., Journé C., Michel J.-B., Jacob M.-P. (2014). Inhibition of ERK1/2 phosphorylation: A new strategy to stimulate elastogenesis in the aorta. Hypertension.

[23] Doyle J.J., Doyle A.J., Wilson N.K., Habashi J.P., Bedja D., Whitworth R.E., Lindsay M.E., Schoenhoff F., Myers L., Huso N. (2015). A deleterious gene-by-environment interaction imposed by calcium channel blockers in Marfan syndrome. eLife.

