# Supplementary material for: Marfan Syndrome Variability: Investigation of the Roles of Sarcolipin and Calcium as Potential Transregulator of FBN1 Expression
Source: Genes (Basel). 2018 Aug 21;9(9):421. doi: 10.3390/genes9090421 (PMC6162465; doi:10.3390/genes9090421)
Supplement: Supplementary file 1 [file genes-09-00421-s001.zip › Figure S4.pdf]

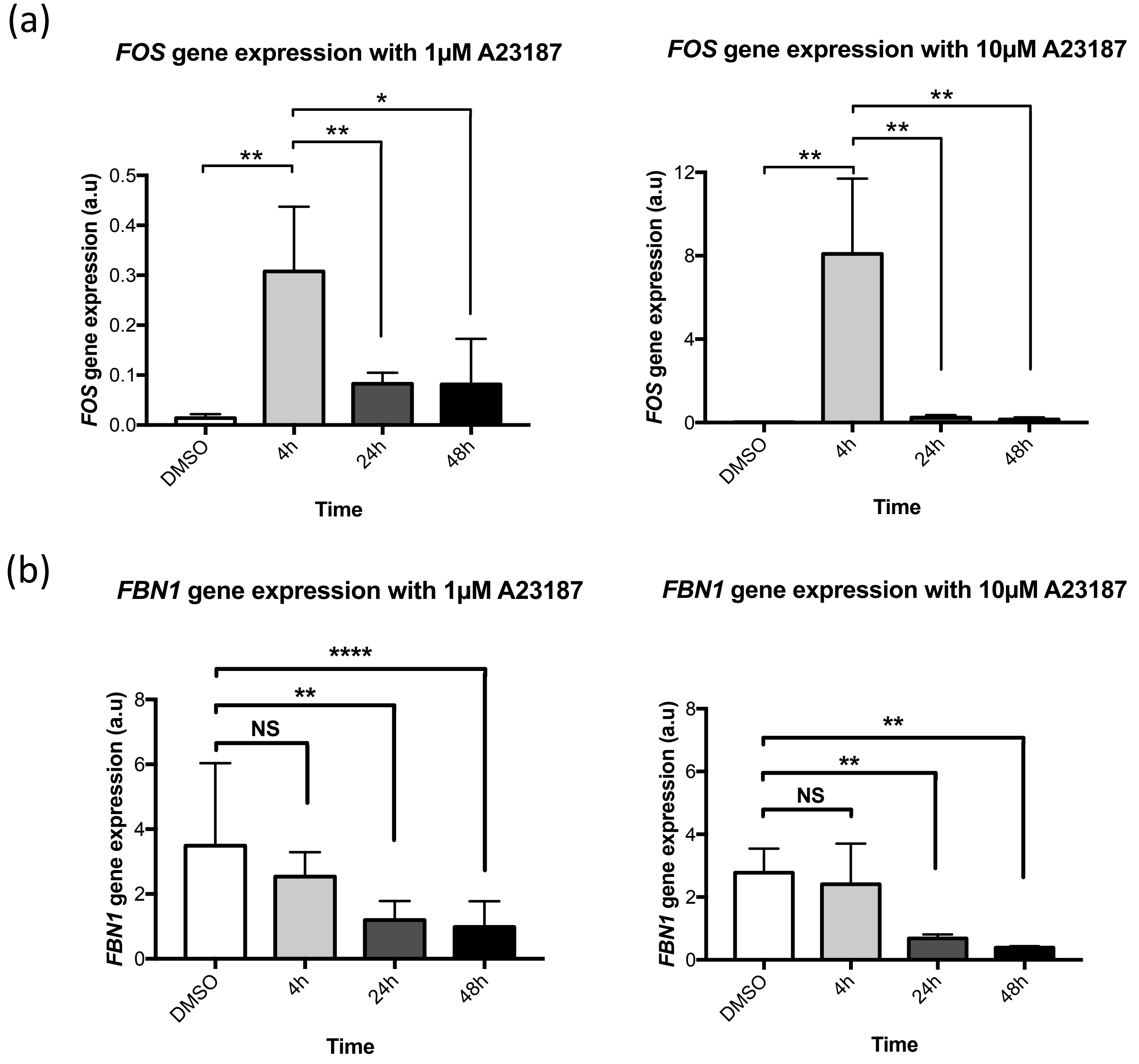

Figure S4. Effect of A23187 on gene expression in control. Control skin fibroblasts were incubated with DMSO (untreated), 1µM or 10µM of A23187 for 4h, 24h, and 48h. (a) *FOS* gene expression (b) *FBN1* gene expression after treatment with A23187

*[NS] p-value > 0.05; **p-value < 0.01; ***p-value < 0.001; ****p-value < 0.0001*
