# Supplementary material for: Marfan Syndrome Variability: Investigation of the Roles of Sarcolipin and Calcium as Potential Transregulator of FBN1 Expression
Source: Genes (Basel). 2018 Aug 21;9(9):421. doi: 10.3390/genes9090421 (PMC6162465; doi:10.3390/genes9090421)
Supplement: Supplementary file 1 [file genes-09-00421-s001.zip › Table S1.pdf]

Name	Foward	Reverse
FBN1	AGTCGGGCCAAGAGAAGAGGCG	TCCATCCAGGGCAACAGTAAGCAT
FOS	AACCGCCACGATGATGTTCT	TCTGCGGGTGAGTGGTAGTA
GAPDH	GTCGCCAGCCGAGCCACATC	CCAGGCGCCCAATACGACCA
SNX7-ps1	CCTTACTACATGGGAACCATTC	GGACATGGCAGATCCAGAAA
SLN	GCTCAAGTTGGAGACAGCGA	CGTGTGGGTTGAAGGGATGT
SNX7	CCCTGAAAGCAGATTGGGAG	GTGAAGGTTGGTCTGTGATGTAAG
SDHA	AAGGGCTCCGACTGGCTGGGG	TTTCTAGCTCGACCACGGCGGC

Table S1: Primers sequences for gene expression quantification.
