# Supplementary material for: Marfan Syndrome Variability: Investigation of the Roles of Sarcolipin and Calcium as Potential Transregulator of FBN1 Expression
Source: Genes (Basel). 2018 Aug 21;9(9):421. doi: 10.3390/genes9090421 (PMC6162465; doi:10.3390/genes9090421)
Supplement: Supplementary file 1 [file genes-09-00421-s001.zip › Figure S1.pdf]

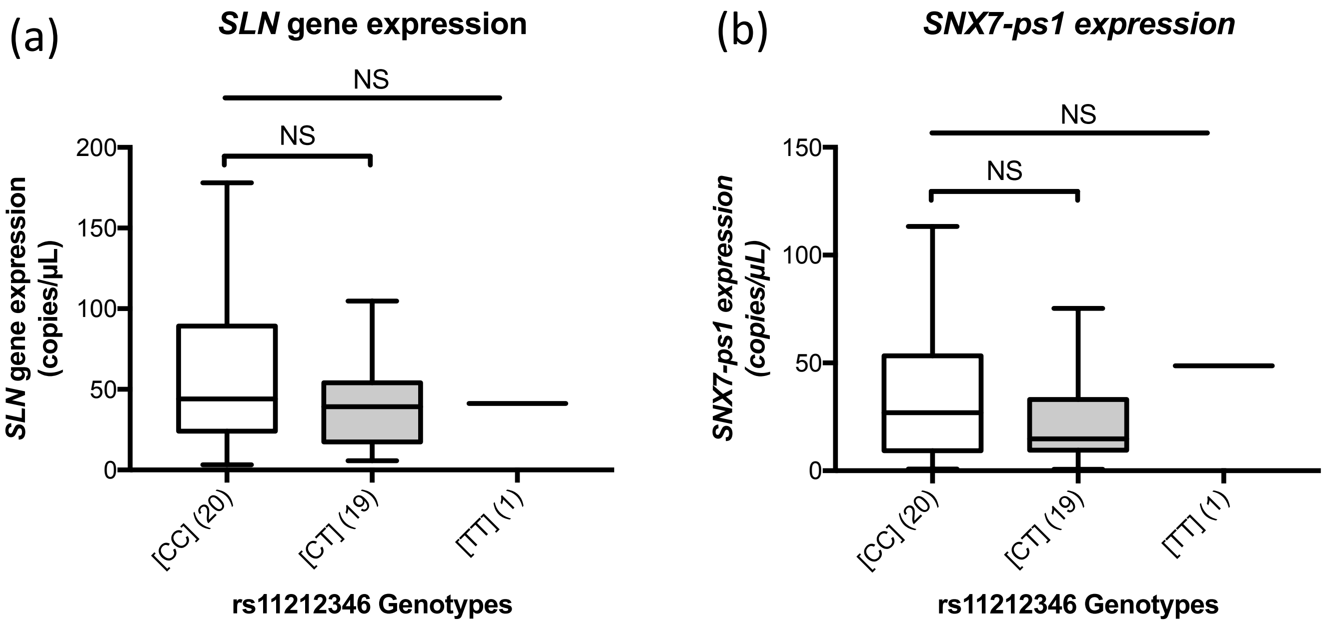

Figure S1. Boxplot of *SLN* and *SNX7-ps1* expression according to rs11212346 genotypes. (a) *SLN* gene. No difference of expression is observed between the three groups (p = 0.30) and between [CC] and [CT] group (p = 0.16) (b) *PSNX7* gene. No difference of expression is observed between the three groups (p = 0.29) and between [CC] and [CT] group (p = 0.50). *[NS] : Not Significant*
