# Supplementary material for: Marfan Syndrome Variability: Investigation of the Roles of Sarcolipin and Calcium as Potential Transregulator of FBN1 Expression
Source: Genes (Basel). 2018 Aug 21;9(9):421. doi: 10.3390/genes9090421 (PMC6162465; doi:10.3390/genes9090421)
Supplement: Supplementary file 1 [file genes-09-00421-s001.zip › Figure S2.pdf]

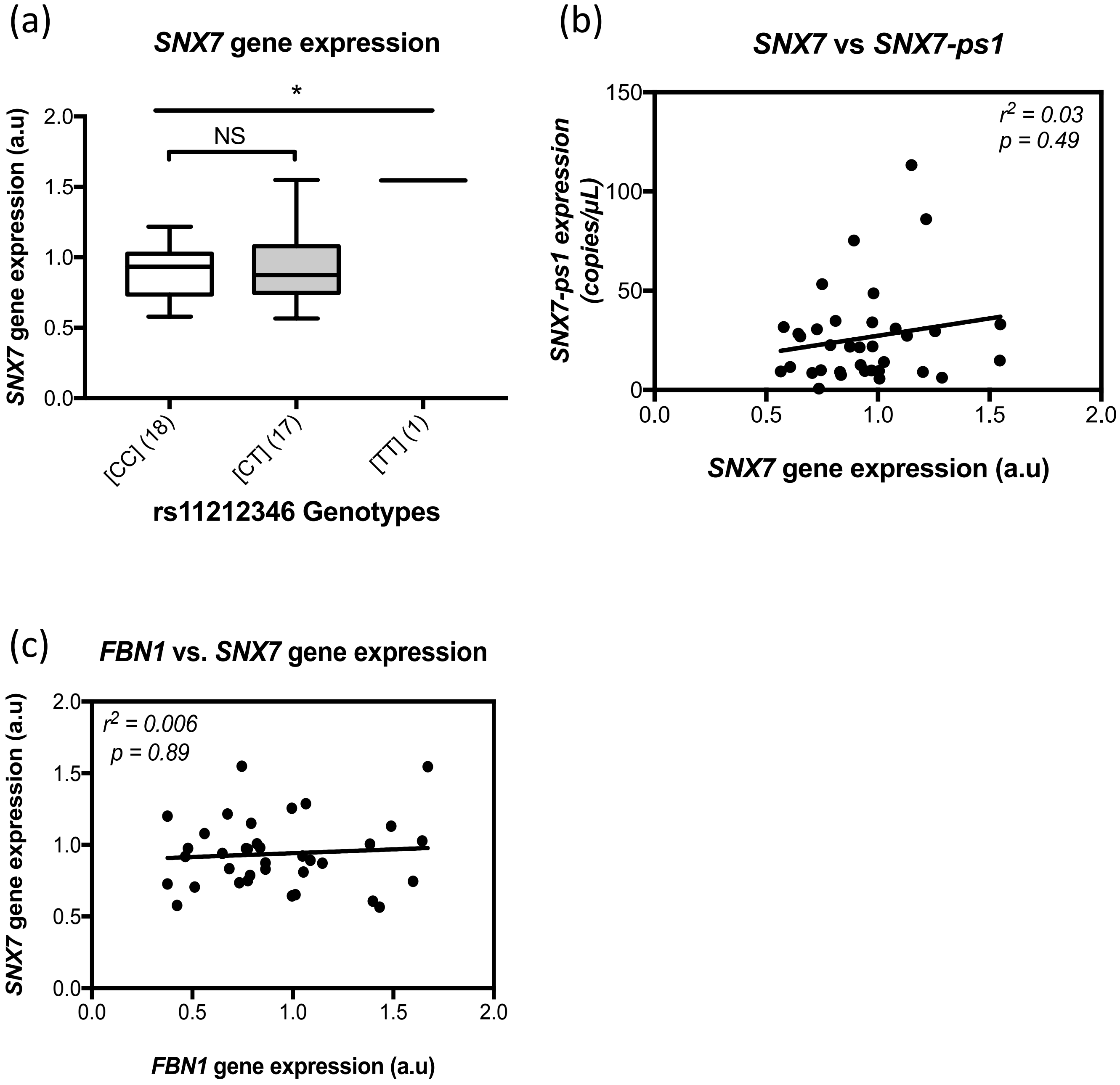

Figure S2. Exploration of the antisense hypothesis for *SNX7-ps1* (a) Boxplot of *SNX7* expression according to rs11212346 genotypes (p-value = 0.033) (b) Correlation plot between *SNX7* and *SNX7-ps1* (c) Correlation plot between *SNX7* and *FBN1* gene expression. No correlation of expression was observed. *[NS] – Not Significant; [*] – p-value < 0.05.*
