# Supplementary material for: Marfan Syndrome Variability: Investigation of the Roles of Sarcolipin and Calcium as Potential Transregulator of FBN1 Expression
Source: Genes (Basel). 2018 Aug 21;9(9):421. doi: 10.3390/genes9090421 (PMC6162465; doi:10.3390/genes9090421)
Supplement: Supplementary file 1 [file genes-09-00421-s001.zip › Figure S3.pdf]

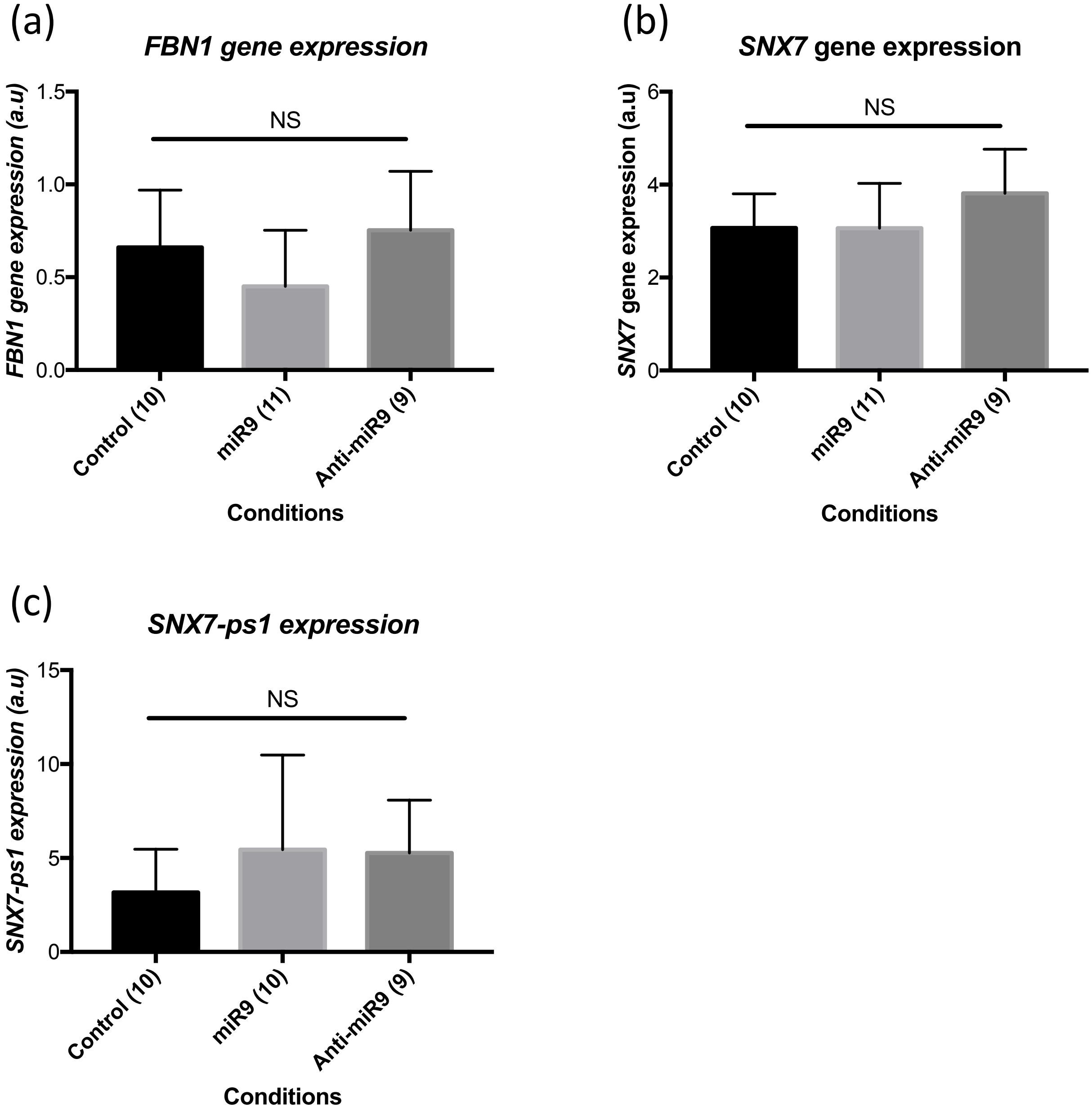

Figure S3. Effect of miR9 on gene expression. (a) *FBN1* gene expression. No significant difference between the three conditions (p-value = 0.07) (b) *SNX7* gene expression. No significant difference between the three conditions (p-value = 0.11) (c) *SNX7-ps1* gene expression. No significant difference between the three conditions (p-value = 0.24).
[miR9] – synthetic miR9; [Anti-mir9] – siRNA targeting mir9; [NS] Not significant
